# Increased phototoxic burn tolerance time and quality of life in patients with erythropoietic protoporphyria treated with afamelanotide – a three years observational study

**DOI:** 10.1186/s13023-020-01505-6

**Published:** 2020-08-18

**Authors:** Jasmin Barman-Aksözen, Michèle Nydegger, Xiaoye Schneider-Yin, Anna-Elisabeth Minder

**Affiliations:** 1 Institute of Laboratory Medicine, Stadtspital Waid und Triemli, Zurich, Switzerland; 2 Institute of Anesthesia and Intensive Medicine, Stadtspital Waid und Triemli, Zurich, Switzerland; 3 Department for Endocrinology, Diabetology, Porphyria, Stadtspital Waid und Triemli, Zurich, Switzerland

**Keywords:** Erythropoietic protoporphyria, Phototoxic reaction, Pain, Phototoxic burn tolerance time, Afamelanotide

## Abstract

**Background:**

Erythropoietic protoporphyria (EPP) is an ultra-rare genetic disorder (prevalence 1:150`000) characterized by instant painful phototoxic burn reactions in skin exposed to visible light. Afamelanotide is the first clinically tested therapy effectively increasing the time EPP patients can spend in direct sunlight without developing symptoms and reducing the number and severity of phototoxic reactions.

**Objectives:**

We report our data on real-world effectiveness of afamelanotide treatment in EPP and its phototoxic burn protection factor (PBPF).

**Methods:**

We analysed clinical data collected between 2016 and 2018 in the Swiss EPP cohort (*n* = 39) on maximum phototoxic burn tolerance time (PBTT), i.e., maximum time spent in sunlight without phototoxic reaction, severity of phototoxic reactions as assessed by an 11-point Likert-type visual analogue scale (VAS), with 0 being no pain and 10 being the worst possible pain, and Quality of Life (QoL), as assessed with an EPP-specific instrument.

**Results:**

Before treatment, the PBTT was median 10 min (IQR 5–20). Under treatment, PBTT increased to median 180 min (IQR 120–240). Individual PBPF increased 1.8- to 180-fold (full range, median 15). The pain severity of the worst phototoxic reaction before treatment was median 10 and under treatment median 6 (IQR 3–7). QoL at the end of the observation period in 2018 (with all the assessed patients under treatment) was 81.4% (IQR 69.4–93.4, *n* = 34). A 97.4% treatment adherence rate was observed.

**Conclusion:**

Treatment of EPP patients with afamelanotide is highly effective under real-world conditions. We suggest PBTT as a clinical meaningful endpoint in further clinical trials.

## Introduction

Erythropoietic protoporphyria (EPP) is an ultra-rare inborn error of metabolism (prevalence 1:150′000) characterized by an excess production and the accumulation of the phototoxic heme precursor protoporphyrin IX (PPIX) during erythropoiesis [1–3]. From early childhood on, EPP patients suffer from phototoxic reactions, i.e., burn-like injuries involving the endothelial layer and basement membranes of the subpapillary capillaries in the dermis of the skin and all light exposed tissues [4, 5]. These phototoxic reactions start within minutes of light exposure and are associated with severe pain, which can last for several days and does not respond to analgesics [6–10]. Often, there are no visible skin alterations [11]. There is a considerable disease heterogeneity within EPP, probably dependent on biological and environmental factors [8, 9]. In order to avoid the incapacitating symptoms, EPP patients are forced to restrict their exposure to visible light to a minimum. Despite their conditioned light avoidance behaviour, the patients develop painful burns in everyday situations, when they are unable to avoid sunlight, for instance, when waiting at the bus stop, and even when crossing a street. Also, light passing through windows affects patients with EPP. Moreover, a significant proportion of EPP patients do not tolerate artificial light [8, 12]. These constraints have a negative impact on all daily activities leading to impaired career and life choices, social isolation, anxiety, depression and overall decreased Quality of Life (QoL) [6, 10, 12–15]. Because PPIX absorbs energy from the visible light range, ultraviolet radiation filters like sunscreens do not provide any protection. Until recently, no effective therapy to either prevent or treat phototoxic reactions in EPP existed [16, 17].

Afamelanotide (Scenesse®, Clinuvel Pharmaceuticals Ltd.) is the first clinically tested therapy that significantly increases the time patients can spend outdoors in sunlight without developing painful phototoxic reactions and it effectively reduces the frequency and severity of these phototoxic reactions [17, 18]. Afamelanotide is an analogue of the endogenous alpha-melanocyte stimulating hormone. It moderately increases eumelanin synthesis and has strong anti-inflammatory and anti-oxidative properties [19–21].

Since 2006, afamelanotide has been tested in two phase II and three phase III randomized, controlled clinical trials (RCTs), collectively including 347 EPP patients, all showing significant results in their respective endpoints [17, 22]. The endpoints in the clinical trials included time to onset of symptoms during photo-provocation tests, time spent in direct sunlight as assessed in diaries, number and severity (“pain”) of phototoxic reactions as assessed by an 11-point Likert-type visual analogue scale (VAS), and Quality of Life (QoL) with a disease specific, partly validated instrument, the “EPP-QoL”. In the pivotal clinical trial CUV039, the primary endpoint was defined as the time in direct sunlight on days without phototoxic reactions (“pain”). However, details on variable environmental factors, like the overall time spent outdoors on a given day, or the weather conditions, were not included. Nonetheless, per person, a significant difference of 28.6 h (median) additional pain free sunlight exposure time compared to the placebo-treated control group was demonstrated in the 180-day study period [17]. From patient reports [23, 24] and according to our clinical experience, however, these results may underestimate the real-life benefit of the treatment.

In this article, we are reporting our real-world experiences from the Swiss EPP cohort from 2016 to 2018 (*n* = 39). We are providing long-term data on the effectiveness of afamelanotide by using a new endpoint “phototoxic burn tolerance time” (PBTT), i.e., the reported maximum time the patients are able to expose themselves to sunlight without experiencing a phototoxic reaction, as well as data on the severity of a phototoxic episode (i.e., maximum VAS), QoL, and treatment adherence.

## Patients and methods

### Patients

Between 2016 and 2018, 39 adult EPP patients with residency in Switzerland received treatment with afamelanotide at our centre. Written informed consent was obtained from all patients after receiving written study information on the use of their clinical data. The study was approved by the ethic review board.

### Data source

We analysed the data available from our clinical outpatient visits retrospectively. Our clinical notes included a baseline documentation on medical history (including data back until 1993), the disease specific EPP-QoL questionnaire, and a clinical course form; the latter were both completed during every appointment.

### Frequency and duration of the treatment with afamelanotide

Afamelanotide is administered subcutaneously as a 16 mg slow release implant formulation with a therapeutic effect of approximately 60 days [21, 25]. Of the 39 EPP patients in this study, six started their treatment during the observation period and 33 were already treated with afamelanotide before 2016, i.e., during the clinical trials, compassionate use programs and a special access scheme for therapy reimbursement in Switzerland. The median duration of treatment before 2016 was 3.5 years (IQR 2.8–8.5 years). Some of these patients had been continuously on treatment since the first phase II clinical trial in 2006, and received up to six doses per year. Twenty patients (51%) had treatment interruptions of various durations due to the denial of reimbursement during 2016 and 2017 (treatment interruption median 413 days, IQR 287–642).

For the present analysis the frequency of dosage with the exact dates of the implant and treatment intervals (as calculated in days after last implant) were obtained from the clinical notes. The number of annual doses was dependent on the medical need (e.g., symptoms during winter caused by artificial light, necessity to travel to sunny areas in winter, variability of individual patients’ tolerance to light) and, in certain cases, restricted by health insurance companies reimbursing a lower number of doses than medically indicated.

### Treatment adherence rate

Based on the number of patients requesting a treatment continuation with afamelanotide between 2016 and 2018, we calculated the treatment adherence rate. Compelling reasons for a withdrawal, such as an intended pregnancy, relocation to another country, as well as cessation of reimbursement by the health insurer or other financial restrictions, were excluded for the calculation of the treatment adherence rate.

### Assessment of sunlight tolerance and severity of phototoxic reactions

We documented the maximum time a patient reported to be able to spend in sunlight until a phototoxic reaction develops before treatment. In addition, we asked about the maximum symptom severity the patient had ever experienced using an 11-point Likert-type visual analogue scale (VAS), with VAS 0 being no pain and VAS 10 being the worst imaginable pain. The VAS were rounded to integral numbers.

Under treatment, we documented the maximum time the patient spent in sunlight on a sunny day, the number of phototoxic reactions, as well as the severity and duration of the worst phototoxic reaction during the preceding 2 months. As afamelanotide has a therapeutic effect for approximately 60 days, in order to determine the maximum VAS under treatment and not to miss any data due to varying treatment appointments, we used the maximum VAS occurring within 80 days after the last implant of afamelanotide.

### Phototoxic burn tolerance time and phototoxic burn protection factor

Phototoxic burn tolerance time (PBTT) was defined as the maximum time the patient spent in sunlight without suffering from a phototoxic reaction. From these data, for each patient a phototoxic burn protection factor (PBPF) was calculated by dividing the PBTT under treatment by the PBTT before treatment.

### Quality of life

The assessment of QoL was based on the disease specific EPP-QoL questionnaire (12-item version) [26]. In total, 266 EPP-QoL questionnaires from 35 patients were collected in the observational period. The results of the questionnaires were expressed as percentage of maximum, with 100% being the best and 0% the worst possible QoL.

### Statistical analysis

Statistical analysis was performed using median values and interquartile range (IQR)/full range, Wilcoxon paired t-test for pairwise comparison and the Kruskal-Wallis test with the Tukey-Kramer extension for the comparison of more than two groups. For correlations, we used a Kendall’s tau test. A two-sided *p*-value < 0.5 was considered statistically significant.

## Results

### Quality and validity of the study data

As this study relies on retrospectively collected information, we first determined whether the data results in meaningful observations (for full information see supplement 1). Based on these analyses, we concluded that the data source used for the presented study provides reliable results.

### Effect on phototoxic burn tolerance (PBTT) under treatment with afamelanotide

**Fig. 1:**
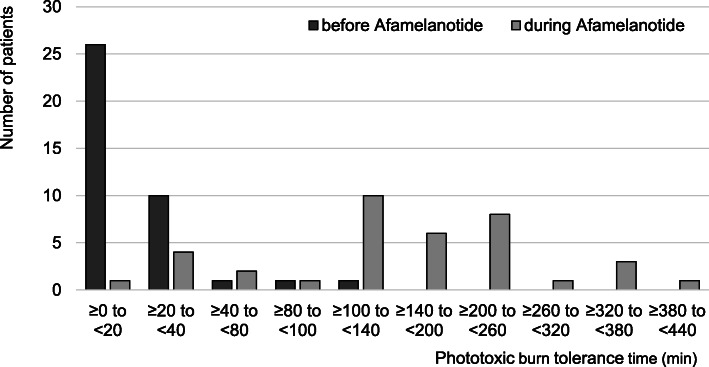
Maximal phototoxic burn tolerance time (PBTT) before and under treatment with afamelanotide. Thirty-nine patients were assessed before first dose, and 37 during treatment with afamelanotide

In our cohort, EPP patients without treatment reported a median of 10 min as the maximum time they can spend in sunlight in the absence of a phototoxic reaction, i.e., PBTT (IQR 5–20 min, range 2–120 min, *n* = 39). Under treatment with afamelanotide, the PBTT significantly increased to a median of 180 min (IQR 120–240 min, range 15–420 min, *n* = 37 (2 patients with missing data); *p* < 0.0001) (see Fig. 1).

As shown in Fig. 1, as there is an overlap of PBTT before and during treatment with afamelanotide, we excluded that only a subgroup of patients increased their PBTT. In our cohort, all patients under treatment increased their individual PBTT and afamelanotide provided a median PBPF of 15 (IQR 6–36, range 1.8–180, *n* = 37; *p* < 0.0001). The extent of the increased PBTT under treatment was independent of the reported PBTT before treatment (Kendall’s tau = 0.105, *n* = 37; *p* = 0.41).

### PBTT in relation to the time span since the preceding afamelanotide dose

**Fig. 2:**
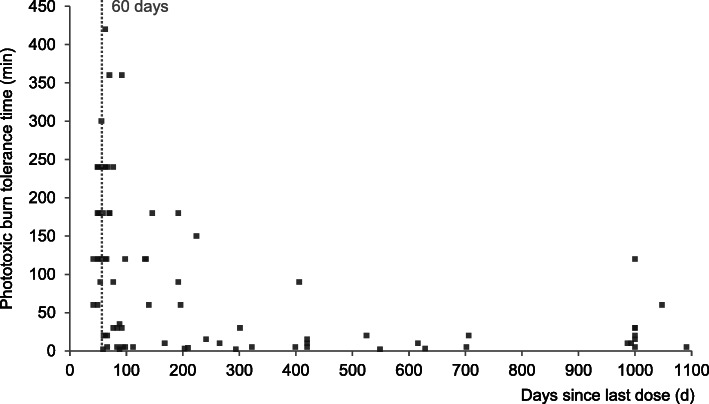
Correlation of PBTT and days since the last dose of afamelanotide. The first afamelanotide dose a patient ever received was defined as being equal to 1000 days of treatment interruption. PBTT inversely and significantly correlated to the time span to the last dose of afamelanotide (Kendall’s tau = − 0.382, *n* = 82; *p* < 0.0001)

We investigated whether the time span since the preceding implant influenced the PBTT in our cohort. Indeed, PBTT inversely and significantly correlated to the time span of the last dose of afamelanotide (Kendall’s tau = − 0.382, *n* = 82; *p* < 0.0001). As evident in Fig. 2, PBTT nearly exponentially decreased, if the time interval between the doses exceeded the recommended 60 days.

### Decrease of the severity of phototoxic reactions despite longer sun exposure time

**Fig. 3:**
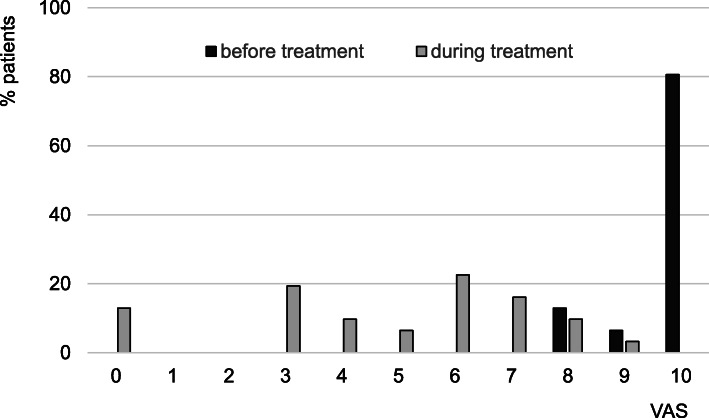
Number and severity of maximal phototoxic episodes before and during treatment with afamelanotide. For the number and severity of phototoxic episodes during treatment we assessed the preceding 2 months and considered only values, when the time interval since the last dose did not exceed 80 days

**Fig. 4:**
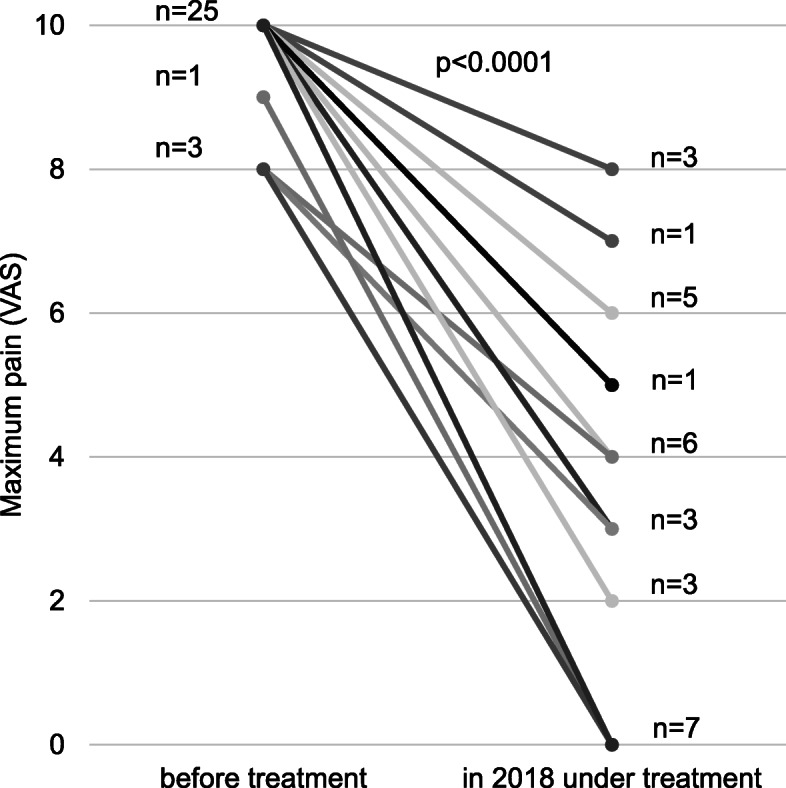
Maximum pain severity (VAS) before treatment and under continuous treatment in 2018 (i.e. without involuntary treatment interruptions). The lines represent the maximal VAS of individual patients. Only values, when the time interval since the last dose did not exceed 80 days, were considered. Median pain severity before treatment VAS 10, in 2018 under treatment median VAS 4 (IQR 2–6)

Without an effective therapy, we would expect that the observed increased sunlight exposure under treatment would aggravate the number and severity of phototoxic reactions. Before treatment, the maximum reported pain intensity was a median 10 (IQR 10–10, range 8–10, *n* = 36, 3 patients with missing baseline data) and 29 (80.6%) of our patients had at least once in their lifetime suffered from a pain severity of 10 (see Figs. 3 and 4). Under treatment, the median maximal individual pain severity was significantly lower (median 6, IQR 3–7, range 0–9, *n* = 31 (8 patients with missing data); *p* < 0.0001) and each patient had a lower pain score than reported before. This effect was even more pronounced when only analysing the worst episode in the year 2018 when all patients, including those with treatment interruptions in 2016/2017, were treated (median pain severity 4, IQR 2–6, range 0–8, *n* = 29; *p* < 0.0001) (see Fig. 4).

### Effect of the afamelanotide treatment on quality of life

During the observation period, six patients started the treatment with afamelanotide. At baseline, i.e. the QoL before the first treatment, they showed a median QoL of 49.1% (IQR 16.4–65.5). When assessing all patients under treatment at the end of the observation period in 2018, the QoL was 81.4% (IQR 69.4–93.4, *n* = 34). The QoL in continuously treated patients remained unchanged throughout the observation period (median QoL 66.7–83.3%, *p* = 0.24). The first QoL in patients after an interrupted treatment was significantly lower compared to that of patients who were treatment-naïve (median 16.7% vs. 49.1%, *p* = 0.0473, *n* = 26), but under treatment again reached levels also found in individuals with continuous treatment (median 84.8%, IQR 66.9–94%).

### High adherence rate to the afamelanotide treatment

During the observational period from 2016 to 2018 two out of 39 patients stopped the treatment. One patient because of an intended pregnancy, which is a compelling reason for a treatment interruption, and one because of an adverse event not related to afamelanotide, resulting in a 97.4% adherence rate to afamelanotide for non-compelling reasons. According to their medical needs and varying reimbursement decisions in 2018 the patients in the Swiss cohort received between 1 and 6 doses per year (median 4, IQR 4–5).

## Discussion

Measuring treatment effects in EPP is confounded by variables such as seasonal and meteorological conditions, as well as indoor activities, especially the work situation, which limit a regular sunlight exposure. As the endpoints used in the RCTs were based on daily recordings of sunlight exposure times, these recordings may therefore only inadequately capture the disease-specific limitations of light exposure in EPP. We considered the maximum sunlight exposure without a phototoxic burn (i.e., PBTT) as a measure of sunlight tolerance less confounded by the above mentioned influences. PBTT is easily assessed in real-life situations and seems to reflect the reported real-world experience of patients [23, 24] more adequately than any previously applied measurement method. In our cohort, the patients under treatment were able to expose themselves to sunlight significantly longer without developing phototoxic burn reactions than without treatment. Moreover, this newly introduced endpoint enables the calculation of a phototoxic burn protection factor (PBPF), which can be applied to any type of treatment. In our cohort, afamelanotide provided a median PBPF of 15. Further and as evident from Fig. 2, PBTT rapidly decreased if the time interval between the doses exceeded the duration of the therapeutic effect of one implant, which, again, illustrates the protective effect of afamelanotide.

Afamelanotide is not a causative treatment and phototoxic reactions after prolonged exposure to visible light are still possible. However, in our cohort, the pain intensity under treatment decreased despite all treated patients considerably increasing their time in sunlight. The combined improvement of PBTT and pain severity likely explain the consistently positive effects on QoL during afamelanotide we have also documented in our patients. It is especially noteworthy that the baseline QoL in patients with an interrupted treatment was significantly lower compared to that of patients who were treatment-naïve, but under treatment again reached levels also found in individuals with continuous treatment. This indicates that patients with a lifelong disease initially overestimate their QoL, as they have never fully experienced the meaning of a healthy, normal life before [27]. Therefore, any analysis of QoL data in patients with a chronic condition with early childhood onset should appreciate this phenomenon.

All these results and the rapid decrease of PBTT with a treatment interval exceeding 60 days as well as the significantly lower QoL after a treatment interruption not only underline the effectiveness of afamelanotide in EPP but also highlight the need for an individualized dosage regimen according to the medical needs in this patient population with considerable disease heterogeneity.

We further found a treatment adherence rate of 97.4% in our cohort, which is in line with the treatment adherence rate of 98% determined in the post-authorization safety and effectiveness study conducted in the Dutch EPP patient cohort (*n* = 117) and an eight-year observational study on 115 EPP patients with access to the afamelanotide treatment through compassionate use and special access schemes in Italy and Switzerland [26, 28]. This treatment adherence rate exceeds the average adherence to long-term therapies, typically ranging between 43 and 78% [29, 30] and a higher adherence rate could be indicative of a positive treatment experience and less adverse events [29, 31]. So far, no serious adverse events or safety issues related to the use of afamelanotide were identified [26, 32, 33].

The limitations of our study lie in the retrospective and observational nature of the study design and the limited number of participants. However, such a design is considered suitable in ultra-rare conditions such as EPP and is often used in post-admission surveillance for noxious side effects [28, 34].

## Conclusion

In conclusion, our real-life data suggest that, so far, the effectiveness of afamelanotide may have been underestimated and may go beyond the reported benefit in the RCTs. In this regard, we suggest PBTT and PBPF as a reasonable, clinically meaningful and easily applicable parameter to evaluate the real-world effectiveness of any treatment in EPP. Therefore, together with pain severity, PBTT could also be used as an endpoint in future RCTs.

## Supplementary information

**Additional file 1.** Appendix S1: Quality of study data

## Data Availability

The dataset(s) supporting the conclusions of this article are available upon request from the corresponding author.

## Abbreviations

Alpha-MSH: Alpha-melanocyte stimulating hormone
EPP: Erythropoietic protoporphyria
IQR: Interquartile range
PBTT: Phototoxic burn tolerance time
PBPF: Phototoxic burn protection factor
QoL: Quality of Life
VAS: Visual analogue scale
