# Supplementary material for: Increased phototoxic burn tolerance time and quality of life in patients with erythropoietic protoporphyria treated with afamelanotide – a three years observational study
Source: Orphanet J Rare Dis. 2020 Aug 18;15:213. doi: 10.1186/s13023-020-01505-6 (PMC7437008; doi:10.1186/s13023-020-01505-6)
Supplement: Supplementary file 1 — Additional file 1. Appendix S1: Quality of study data [file 13023_2020_1505_MOESM1_ESM.docx]

**Appendix S1:** Quality of study data

Protoporphyrin IX (PPIX) concentrations in peripheral blood samples were measured with the identical in-house method throughout the whole time period [1].

PPIX acts as a photosensitizer in the skin, and a higher blood level of PPIX has been described to weakly correlate to a higher degree of light intolerance, or a shorter phototoxic burn tolerance time (PBTT) [2]. In accordance with these previous studies, we also found a significant inverse correlation of the mean erythrocyte PPIX concentrations prior to the first afamelanotide dose and the PBTT prior to treatment in the Swiss cohort (see figure S1). Further, as expected, the severity of the phototoxic reactions and their duration strongly correlated (Kendall’s tau = 0.736, p<0.0001, n=283). Moreover, the number of phototoxic reactions a patient suffered from during a treatment period correlated with their severity (Kendall’s tau = 0.706, p<0.0001, n=289). In addition, the PBTT during treatment was inversely correlated with the severity of the phototoxic burns (Kendall’s tau = -0.218, p=0.0115, n=76). As presumed, these results indicate that more strongly affected EPP patients indeed have a lower PBTT. Based on these observations we can conclude that the data source used for this retrospective study provides reliable results

.

**Figure S1:** Phototoxic burn tolerance time (in min) before afamelanotide in relation to protoporphyrin IX (PPIX) concentrations (µmol/L)

**References:**

1. Minder EI, Schneider-Yin X: **Porphyrins, Porphobilinogen, and d-Aminolevulinic Acid**. In: *Laboratory Guide to the Methods in Biochemical Genetics.* Edited by Blau N, Duran M, Gibson KM. Berlin-Heidelbarg: Springer; 2008: 751-780.

2. Balwani M, Naik H, Anderson KE, Bissell DM, Bloomer J, Bonkovsky HL, Phillips JD, Overbey JR, Wang B, Singal AK *et al*: **Clinical, Biochemical, and Genetic Characterization of North American Patients With Erythropoietic Protoporphyria and X-linked Protoporphyria**. *JAMA Dermatol* 2017, **153**(8):789-796.
